# Supplementary material for: Nucleus Accumbens-Associated Protein 1 Binds DNA Directly through the BEN Domain in a Sequence-Specific Manner
Source: Biomedicines. 2020 Dec 14;8(12):608. doi: 10.3390/biomedicines8120608 (PMC7764960; doi:10.3390/biomedicines8120608)
Supplement: Supplementary file 1 [file biomedicines-08-00608-s001.pdf]

biomedicines

Nucleus Accumbens-Associated Protein 1 Binds DNA Directly through the BEN Domain in a Sequence-Specific Manner

Naomi Nakayama, Gyosuke Sakashita, Takashi Nagata, Naohiro Kobayashi, Hisashi Yoshida, Sam-Yong Park, Yuko Nariai, Hiroaki Kato, Eiji Obayashi, Kentaro Nakayama, Satoru Kyo and Takeshi Urano

Supplementary Materials

Table 1. Structural Statistics for NAC1 (322-485).

**NMR restraints**		
Distance restraints		
Total NOE	2364	
Intra-residue	648	
Inter-residue		
Sequential (∣i - j∣ = 1)	640	
Medium-range (1 < ∣i - j∣ < 5)	499	
Long-range (∣i - j∣ ≥ 5)	577	
Hydrogen bonds restraints [a]	248	
Dihedral angle restraints [a]		
φ and ψ	111/111	
χ^1 and χ^2	22/14	
Structure statistics (20 conformers)		
CYANA target function (Å^2)	3.56	
Residual NOE violations		
Number > 0.1 Å	14	
Maximum (Å)	0.59	
Residual dihedral angle violations		
Number > 5º	2	
Maximum (º)	12.20	
AMBER energies (kcal/mol)		
Mean AMBER energy	-7217	
Mean restraints violation energy	29.02	
Ramachandran plot statistics (%) [b]		
Residues in most favored regions	93.0	
Residues in additionally allowed regions	6.0	
Residues in generously allowed regions	0.9	
Residues in disallowed regions	0.1	
Average R.M.S.D. to mean structure (Å) [c]	Region A	Region B
Protein backbone	0.67	0.49
Protein heavy atoms	1.12	1.05

[a] Used only in CYANA calculations. [b] Calculated with RAMPAGE server [1]. [c] Region A: For residues A345-I351, K368-E370, Y378-T380, R381-S390, H395-A404, S434-F447, and E453-A461. Region B: For residues K368-E370, Y378-T380, R381-S390, H395-A404, S434-F447, and E453-A461.

Table S2. Thermodynamic parameters (average of three experiments) for the interactions between oligonucleotides and NAC1 (322-485).

oligonucleotide	Binding event	K_d (μM)	ΔH (kcal/mol)	ΔS (cal/mol/deg)	N
GP1-dsDNA	first	0.16	1.8	37.1	0.77
	second	18.0	-30.4	-80.2	0.32
GP1 mut -dsDNA	first	135	-1162	-3880	< 0.1
	second	0.37	-51570	29.2	1.03
GP1-ssDNA	first	0.2	-11000	-36861	< 0.1
	second	0.46	-11.4	-9.1	1.13
de novo motif 1	single	3.1	2.3	33.0	0.67
de novo motif 2	single	0.83	2.2	35.2	0.87

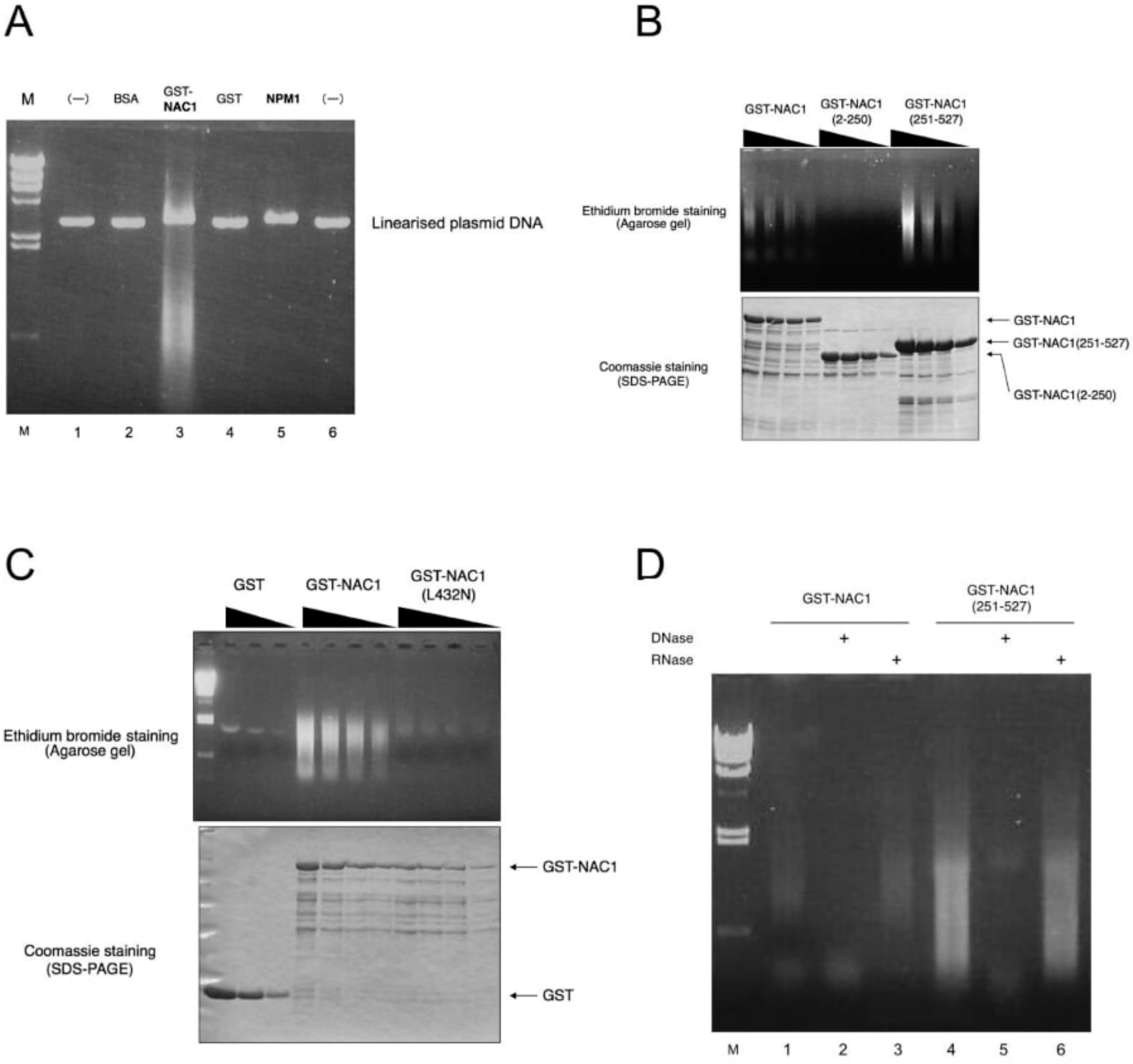

Figure 1. (a) BSA, bacterially expressed and purified GST-NAC1(2-527; full-length), GST alone, and NPM1 as a positive control were each incubated with linearized pBluescript SK II plasmid DNA, followed by gel mobility shift assays. The agarose gel was then stained with ethidium bromide. *Lanes 1* and *6* contain free linearized plasmid DNA. (b) Bacterially expressed and purified GST-NAC1, GST-NAC1 (2-250), and GST-NAC1 (251-527) were each subjected to a bacterial genome carry-over assay. The agarose gel (*upper panel*) and SDS-PAGE gel (*lower panel*) were stained with ethidium bromide

and Coomassie Blue, respectively. (c) Bacterially expressed and purified GST alone, GST-NAC1, and GST-NAC1 (L432N) were each subjected to a bacterial genome carry-over assay. (d) DNase or RNase treatment of carry-over materials. Bacterially expressed and purified GST-NAC1 and GST-NAC1 (251-527) were each treated with DNase or RNase respectively, and then subjected to a bacterial genome carry-over assay.

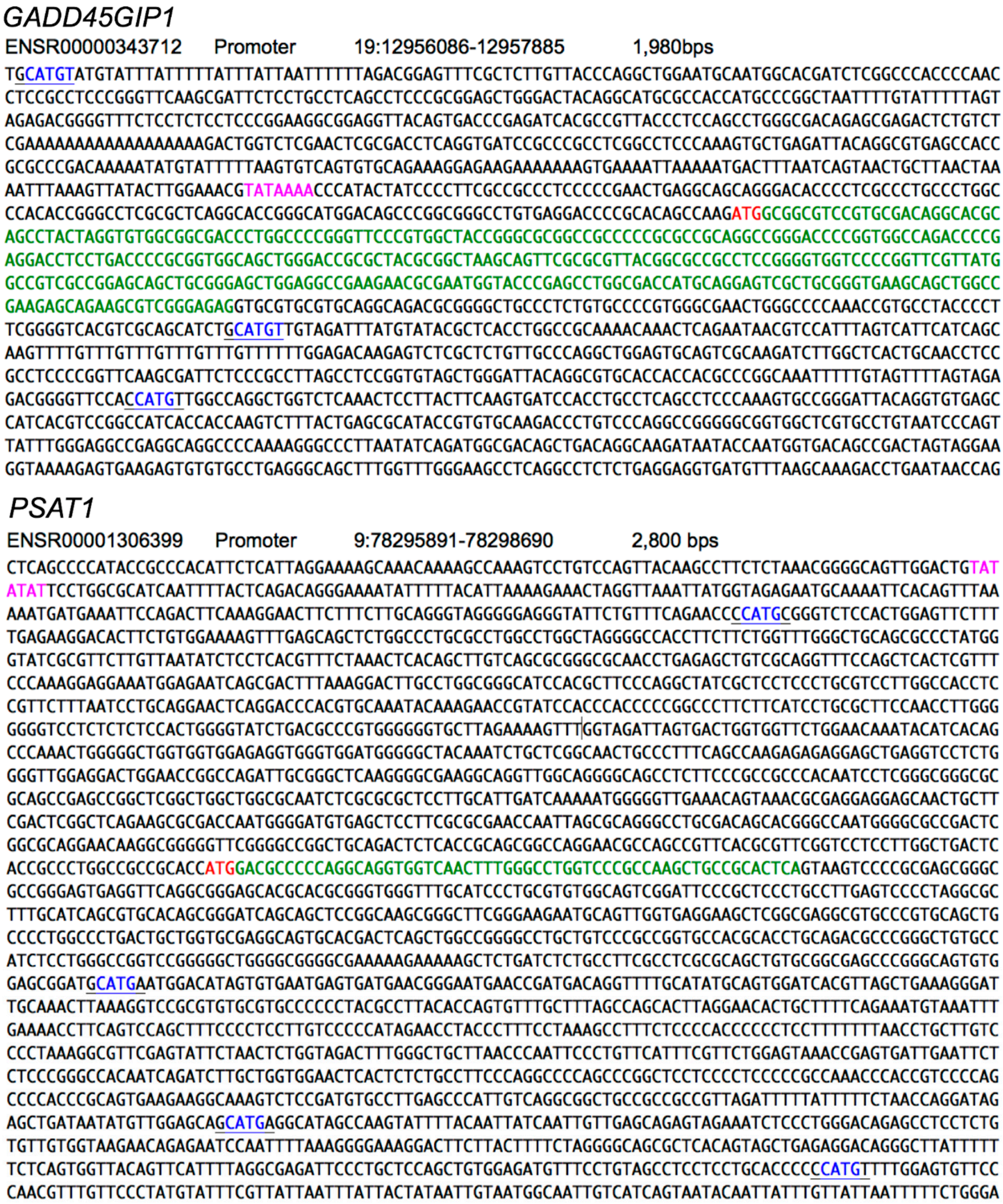

AZU1

ENSR00000341277 Open chromatin 19:827356-828168 814 bps

CCTGGGCAACAAGAGCGAAACTCCGTCTCAAAATAATAATAATAATAATAATAATAATAATCACCCATACAGACGCGCAGGACTCAACGCAGGACTGCCA
GCTCCACTGCCCTGAGCCCCCCGGTGCCAGGCCCAGCGGGCAGTGCTGGAGCCCCAGTGGCTTGGGATGGGGAGACTGGGACTCTGAGCTGGCAATGTTC
CTGGGTGCTGGCTGCATGCCCGCCATGTGCTGGGCTCCGGGATCCACTGGTTCCTGACACCCTCACCTGCCCCTGGGGGTGTGGCCATCTTCTAGAGAGG
GAAACTGAGGATCAGTGCAGAATGTAGGGGGAGCCCAGGCTGGCCCAGGGAGCAGTTGGCGGTGGAGGCCTTGGGCAATTTCCCGTGTTCCCACTGAGTG
GGGCTGTCCCTGGGCCTGGGCGGGGACGCCACCAACTGCCAAGGCCTGTGTATAAGGGCAGCCGCCGCCTTAGCCACAGACCTGCCCCGCCATGACCCGG
CTGACAGTCCTGGCCCTGCTGGCTGGTCTGCTGGCGTCCTCGAGGGCCGGTGAGTGCCTCTCTGTGCCGGTGGTCCCCCATCTGTGCTAGGGCCCGGCTG
CCAGGGCAGAACTCAGACTTAAAGCACAGAGAAGGCAAGCGGCTTGGCCTGGGTCACACAGCCAGCCCGGCCTGGACGATCCCGCGAAAGGCGTGAGGGC
GGACGGTGTGCGGGACTCAGGGGCCCCCTGTCCTCTTAGGGAGTGGGACGATGGGGGAGGGTGGGTCCCCCCGCAGCCCCACTGGGTGGATAGAGCTGAG
GCTGCAGCTTCAC

Figure 2. The promoter region of human *GADD45GIP1*, *PSAT1* and *AZU1* genes harboured **the consensus DNA-binding sequence of NAC1.** The promoter regions (*GADD45GIP1*, NSR00000343712; *PSAT1*, ENSR00001306399; *AZU1*, ENSR00000341277) were predicted by the Ensembl regulatory build database [2]. **The consensus DNA-binding sequences of NAC1 are underlined in blue.** Sequences corresponding to the first methionine codon ATG, first exon, and TATA boxes are highlighted with red, green and magenta characters, respectively.

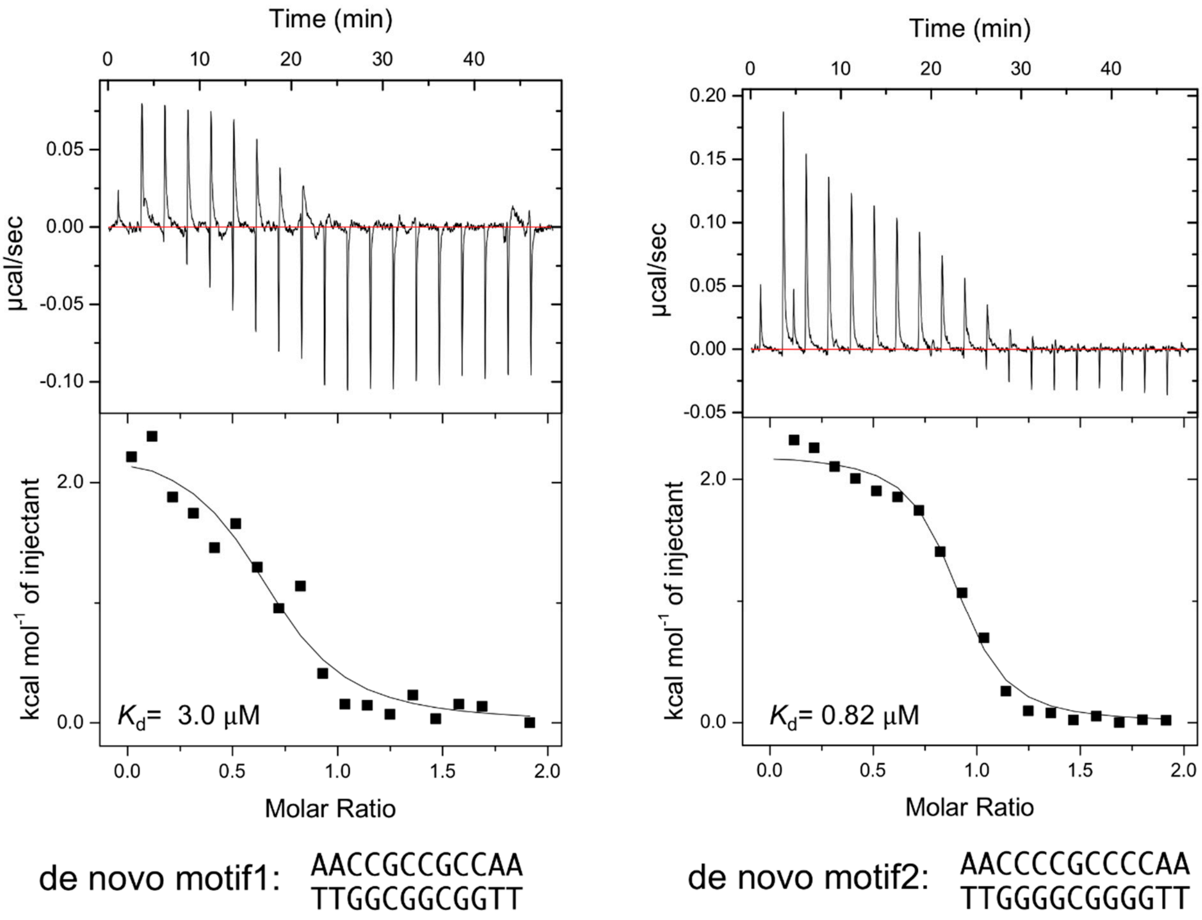

Figure 3. Isothermal titration calorimetry. *Upper panels* are raw titration data plotted as heat (µcal/sec) versus time (min). Each experiment consisted of 28 injections of 10 µl of 50 µM de novo motif1 (*left panel*) or of de novo motif2 (*right panel*) into a solution of 500 µM NAC-1(322-485) at 25°C. The *lower panels* are integrated heat responses plotted as normalised heat per mole of injectant. Smooth curves represent best fits of the data to the equation as described under "Materials and methods" using software provided by the instrument manufacturer. Data shown is representative of three independent experiments.

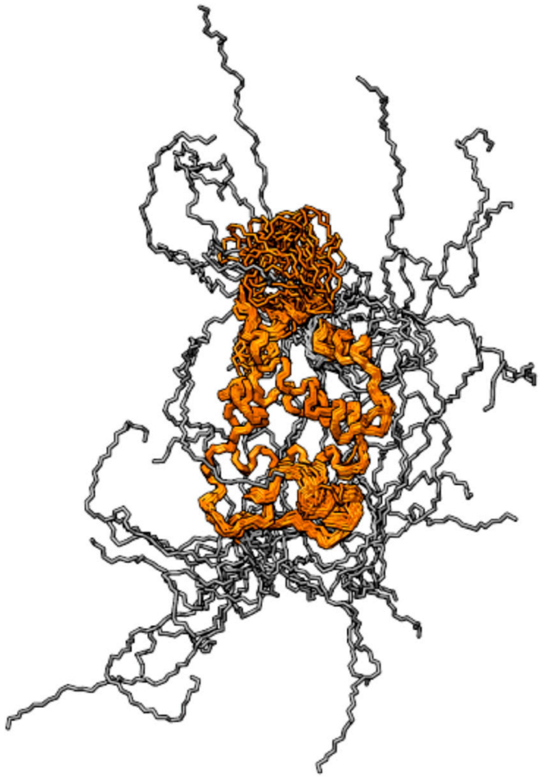

Figure 4. The 20 conformers representing the solution conformation of the BEN domain of NAC1[322-485].

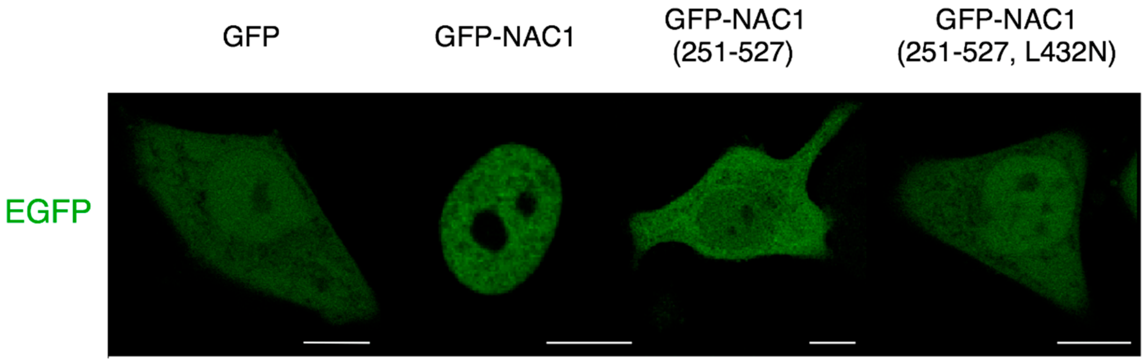

Figure 5. A representative HeLa cell stably expressing GFP, GFP-NAC1, GFP-NAC1 (251-527) or GFP-NAC1 (251-527, L432N). Images were obtained under a 473 diode laser. Bars, 10 μm.

References

1. Lovell, S.C., Davis, I.W., Arendall, W.B., 3rd, de Bakker, P.I., Word, J.M., Prisant, M.G., Richardson, J.S. and Richardson, D.C. Structure validation by Cα geometry: φ,ψ and Cβ deviation. *Proteins* 2003, 50, 437-450.
2. Zerbino, D.R., Wilder, S.P., Johnson, N., Juettemann, T. and Flicek, P.R. The ensembl regulatory build. *Genome Biol.* 2015, 16, 56.
